# Safety of dobutamine or adenosine stress cardiac magnetic resonance imaging in patients with left ventricular thrombus

**DOI:** 10.1007/s00392-023-02317-x

**Published:** 2023-10-16

**Authors:** Lukas D. Weberling, Sebastian Seitz, Janek Salatzki, Andreas Ochs, Jannick Heins, Ailís C. Haney, Deborah Siry, Norbert Frey, Florian André, Henning Steen

**Affiliations:** 1 Department of Cardiology, Angiology and Pneumology, Heidelberg University Hospital, Im Neuenheimer Feld 410, 69120 Heidelberg, Germany; 2 DZHK (German Centre for Cardiovascular Research), Partner Site Heidelberg/Mannheim, Heidelberg, Germany; 3 MVZ-DRZ, Heidelberg, Germany

**Keywords:** CMR, Cardiovascular imaging, Adenosine, Dobutamine, LV thrombus, Stroke

## Abstract

**Background:**

Left ventricular (LV) thrombus formation is a common but potentially serious complication, typically occurring after myocardial infarction. Due to perceived high thromboembolic risk and lack of safety data, stress cardiac magnetic resonance (CMR) imaging especially with dobutamine is usually avoided despite its high diagnostic yield. This study aimed to investigate the characteristics, safety and outcome of patients with LV thrombus undergoing dobutamine or vasodilator stress CMR.

**Methods:**

Patients undergoing stress CMR with concomitant LV thrombus were retrospectively included. Risk factors, comorbidities, and previous embolic events were recorded. Periprocedural safety was assessed for up to 48 h following the examination. Major adverse cardiac events (MACE) 12 months before the diagnosis were compared to 12 months after the exam and between patients and a matched control group. Additionally, patients were followed up for all-cause mortality.

**Results:**

95 patients (78 male, 65 ± 10.7 years) were included. Among them, 43 patients underwent dobutamine (36 high-dose, 7 low-dose) and 52 vasodilator stress CMR. Periprocedural safety was excellent with no adverse events. During a period of 24 months, 27 MACE (14.7%) occurred in patients and controls with no statistical difference between groups. During a median follow-up of 33.7 months (IQR 37.6 months), 6 deaths (6.3%) occurred. Type of stress agent, thrombus mobility, or protrusion were not correlated to embolic events or death.

**Conclusion:**

The addition of a stress test to a CMR exam is safe and does increase the generally high cardioembolic event rate in LV thrombus patients. Therefore, it is useful to support reperfusion decision-making.

**Graphical Abstract:**

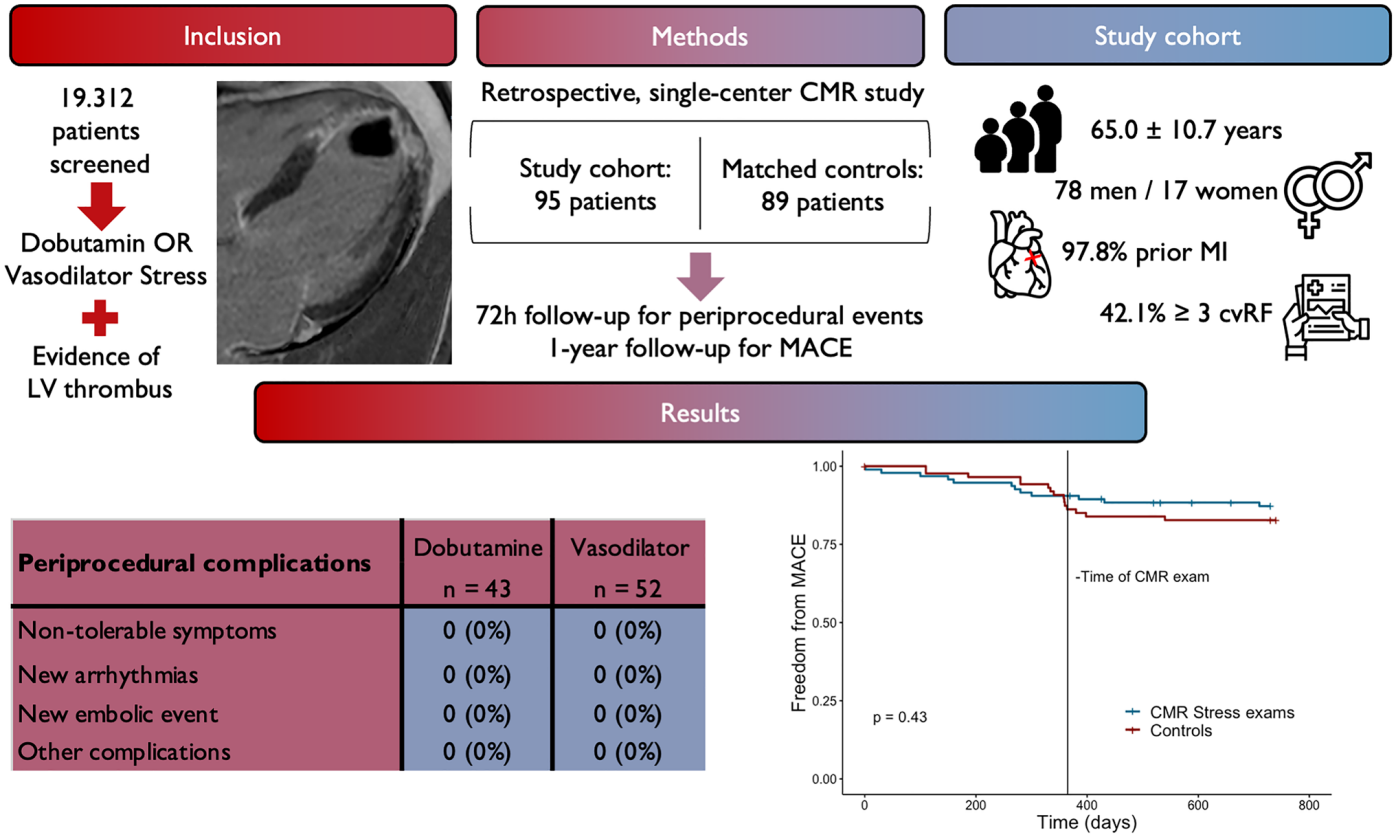

## Background

The formation of a left ventricular (LV) thrombus is a common but often undetected phenomenon following myocardial infarction. Despite significant advancements in primary percutaneous coronary intervention (PCI) treatment, the incidence of LV thrombus after ST elevation myocardial infarction still ranges from 2.7% to 6.3% of all patients [1–5]. Studies utilizing routine cardiac magnetic resonance (CMR) imaging have reported prevalence rates of 8% to 15%, suggesting that many patients with LV thrombi are discharged undiagnosed [6, 7]. Cardiac magnetic resonance (CMR) is considered the gold standard for detecting LV thrombi due to its excellent sensitivity (88%) and specificity (99%), whereas detection with standard transthoracic echocardiography is challenging due to a low sensitivity of 29% to 35% [2, 6, 8, 9].

There is limited data to support prophylactic anticoagulation, even in high-risk patients, due to the associated risk of bleeding [9, 10]. For treating a diagnosed LV thrombus, American and European guidelines recommend oral anticoagulation for 3 to 6 months guided by repeated imaging [11, 12]. This recommended treatment is successful in 62% to 92% of patients [2, 13–15]. While not explicitly stated in the American Guidelines, a majority of recent studies indicate that direct oral anticoagulants are equally effective [11, 13, 15–24].

Systemic embolism is a severe complication of LV thrombus formation with an annual embolic rate of approximately 3.7% [2, 15, 25, 26]. Simultaneously, non-invasive stress testing methods are necessary to assess the hemodynamic relevance of residual coronary stenoses following myocardial infarction [27–32]. Stress CMR using dobutamine or a vasodilator (i.e. adenosine) is an established diagnostic modality recommended by the current guidelines [33, 34]. It possesses both a high diagnostic accuracy non-inferior to invasive fractional flow reserve and an excellent safety profile in the general population [33, 35–37]. Paradoxically, whilst regular exercise generally protects against atherosclerosis, acute exercise is associated with a significant increase in thromboembolic risk for up to 2 h [38]. However, it is unclear how this relates to the periprocedural thromboembolic risk of stress CMR, particularly with high-dose dobutamine infusion. In practice, although not explicitly advised against in the current guidelines, stress CMR in general and dobutamine stress in particular is avoided in patients with LV thrombus [39, 40]. Hence, no data exists on the periprocedural risk and long-term outcome of patients with LV thrombus undergoing stress CMR.

Therefore, this study sought to investigate the characteristics, anticoagulation efficacy, safety, and outcome of patients with LV thrombus undergoing dobutamine or vasodilator stress CMR.

## Methods

### Study population and design

The clinical database was searched for patients undergoing stress CMR (adenosine, dobutamine, or regadenoson) with concomitant LV thrombus between February 2015 and September 2022.

If available, it was noted whether the transthoracic echocardiography reports at the time of CMR (or up to 30 days before or after) detected the LV thrombus. Cardiovascular risk factors such as arterial hypertension, current or former smoking habit, hypercholesterolemia, diabetes mellitus, obesity, and family history of cardiovascular disease were assessed using medical reports. Comorbidities including prior myocardial infarction, prior venous thrombosis, prior arterial embolism, active malignant disease, and known thrombophilia were also assessed. For arterial embolisms, the timing of occurrence relative to the CMR was recorded: > 12 months prior to CMR (no assumed correlation to LV thrombus) or < 12 months before CMR (assumed correlation to LV thrombus). As a control, a sex-, age- and LV ejection fraction-matched control group of patients undergoing a stress-free CMR with concomitant LV thrombus was retrospectively identified in our database. Likewise, prior arterial embolisms < 12 months before CMR (assumed correlation to LV thrombus) were assessed for this group.

### CMR acquisition protocol

CMR imaging was performed at a 1.5 Tesla or a 3 Tesla MRI scanner (Ingenia and Ingenia CX, Philips Healthcare, Best, The Netherlands). Cine images were obtained with a steady-state free precession sequence using retrospective electrocardiographic gating in at least three long-axis planes (two, three, and four-chamber views), as well as a short-axis stack of the whole left ventricle (slice thickness 8 mm) during breath-hold with 35 phases per cardiac cycle. For dobutamine stress, CMR, three long axis and three short-axis planes (basal, midventricular, apical) were acquired before and during dobutamine infusion starting at 10 µg/kg/min and increased by 10 µg/kg/min every step to a maximum of 40 µg/kg/min (20 µg/kg for low-dose vitality testing). In case of an insufficient heart rate response, additional medication with up to 2 mg atropine intravenously was used in the absence of contraindications. For vasodilator stress CMR, an intravenous infusion of 140 or 210 µg/kg/min adenosine for three minutes or a 0,4 mg single injection of regadenoson was followed by a gadolinium-based contrast agent bolus and a three-slice turbo field gradient echo-echo-planar imaging sequence. Patients were able to communicate with the technician or doctor during the exam via intercom and were regularly asked about the occurrence of symptoms like shortness of breath or angina. Late gadolinium enhancement images were acquired 10 min after the contrast agent injection. We used the contrast agent Gadovist^™^ (Bayer AG, Leverkusen, Germany) at a dose of 0.1–0.2 mmol/kg. Image analysis was performed using the IntelliSpace Portal (Philips Healthcare) and cvi42 (Circle Cardiovascular Imaging, Calgary, Canada). Images were verified for the presence of an LV thrombus and analyzed by two independent readers with more than 3 years of clinical CMR experience each. LV wall motion including the presence of a local akinesia or aneurysm (defined qualitatively as an abnormality in the diastolic contour with systolic dyskinesis) was assessed [41]. Each thrombus was classified as either mural (flat and parallel to the endocardial surface) or protruding (projecting into the left ventricular cavity) and mobile (motion of the thrombus independently of surrounding myocardial wall) or non-mobile as described earlier [41]. Global and regional (at the site of thrombus) longitudinal strain was calculated using the feature tracking module of cvi42.

This retrospective study was approved by the ethical commission of our institution (S-151/2019) and followed the declaration of Helsinki.

### Adverse events and follow-up

During the CMR scans, the occurrence of moderate or severe symptoms as well as the occurrence of minor or major adverse events was noted. A minor adverse event was defined as persisting severe symptoms or arrhythmias needing therapy. Major adverse cardiovascular events (MACE) were defined as cardiovascular death or non-fatal thromboembolic event such as stroke, transient ischemic attack, myocardial infarction, or other arterial embolism.

Patients’ follow-up was conducted by telephone interview, hospital follow-up or at their outpatient cardiologist for the occurrence of death from any cause and, limited to 12 months, for the occurrence of MACE. Controls were followed up for 12 months for the occurrence of MACE. Medication with oral anticoagulant before and after CMR was registered for both patients and controls.

### Statistical analysis

Analyses were carried out using the R language and environment for statistical computing (version 4.2.1) with the user interface R Studio (version 2023.06.0/421) [42].

Normal distribution was assessed by using the Shapiro–Wilk test. Parametric variables are given as mean ± standard deviation and non-parametric variables as median with interquartile range.

For the comparison of normally distributed parameters between two groups, the Welch two-sample t-test was used. Non-parametric parameters were tested for differences using the Wilcoxon rank-sum test. A Pearson’s Chi-squared test of independence was employed to test for a correlation between two variables. A Kaplan–Meier estimator was calculated to visualize survival probability. The a priori significance level was set to *p* < 0.05.

## Results

### Study population

19,312 patients in our databank were screened and 95 (78 male/17 female, 65 ± 10.7 years) fulfilled the inclusion criteria. Cardiovascular risk factors were frequent, 40 patients (42.1%) exhibited ≥ 3 risk factors. 93 patients (97.8%) had a history of a prior myocardial infarction, which occurred in the last 6 months before the CMR in 35 (36.8%) of those. In 68 of those patients (73.1%), initial reperfusion therapy had been successful (TIMI 2 in 7 patients, TIMI 3 in 61 patients). In another 15 (16.1%), reperfusion had been attempted but ultimately failed (TIMI 0 in 14 patients, TIMI 1 in 1 patient). After the myocardial infarction diagnosis, of those 83 patients 61 were treated in the form of dual therapy and 22 in the form of triple therapy. Ultimately, in 11 patients (11.8%) a myocardial infarction had not been noticed or diagnosed before, but invasive coronary angiography showed a total (9 patients, TIMI 0) or near total (2 patients, TIMI 1) occlusion of a coronary artery and CMR showed late gadolinium enhancement suggestive of past myocardial infarction. Since troponin values were inconspicuous, no reperfusion therapy was attempted, and no new medication was started in those 11 patients. A relevant portion of patients had an active malignant disease (10.5%), a known thrombophilia (3.2%), or a history of previous arterial (24.2%) or venous (12.6%) thrombosis. For all patients, a complete medical history with all events prior to and up to 48 h after the CMR exam was available. For MACE, a 30 day and 12 month follow-up was available for 94 (98.9%) and 89 (93.7%) patients, respectively. All patient characteristics are given in Table 1.

**Table 1:** Overview of the characteristics of all included patients

Total number of patients	95
Men/Women	78/17 (82.1%/17.9%)
Age (years)	65 ± 10.7
New York Heart Association class	
I	19 (20.0%)
II	49 (51.2%)
III	26 (27.4%)
IV	1 (1.1%)
Canadian Cardiovascular Society grading	
0	24 (25.3%)
1	38 (40.0%)
2	23 (24.2%)
3	10 (10.5%)
4	2 (2.1%)
Cardiovascular risk factors for CAD	
Hypertension	67 (70.5%)
Smoking habit	45 (47.4%)
Hypercholesterolaemia	54 (56.8%)
Diabetes	26 (27.4%)
Family history	31 (32.6%)
Obesity	18 (18.9%)
Medical history	
Prior MI	93 (97.8%)
Culprit lesion: LAD	78 (83.9%)
Culprit lesion: LCX	7 (7.5%)
Culprit lesion: RCA	9 (9.7%)
Peak high sensitivity Troponin T	3095 ng/l (1434; 7797)
MI in the last 6 months	35 (36.8%)
No prior venous or arterial thrombosis	63 (66.3%)
Prior TIA^a^	3 (3.2%)
Prior stroke^a^	8 (8.4%)
Prior LV thrombus^b^	11 (11.6%)
Prior other arterial embolism^a^	4 (4.2%)
Prior pulmonary embolism	5 (5.3%)
Prior deep vein thrombosis	10 (10.5%)
Active malignant disease	10 (10.5%)
Known thrombophilia	3 (3.2%)

*MI* myocardial infarction, *LAD* left anterior descending artery, *RCA* right coronary artery, *LCX* circumflex artery, *TIA* transient ischemic attack

^a^Over 12 months before the current LV thrombus diagnosis

^b^With evidence of disappearance > 12 months prior to current CMR

For the matched control group, 89 patients were identified to match the 89 patients of the study group for whom a 12 month follow-up was available. A 12-month follow-up after CMR and details about embolic events prior to the CMR were available for all controls. As per matching, sex [72 male, 17 female], age (62 ± 13.3 years) and LV ejection fraction (36.7 ± 13.4%) did not differ between the study group and controls. Neither did the rate of anticoagulation prior to (p = 0.84) or after the CMR exam (p = 0.77). The underlying cardiac pathology was an ischemic cardiomyopathy in 61 (68.5%), a dilated cardiomyopathy in 14 (15.7%) and another non-ischemic cardiomyopathy in 14 (15.7%). Cardiovascular risk factors were equally common with hypertension in 51 (57.3%), a smoking habit in 50 (56.2%), hypercholesterolaemia in 35 (39.3%), diabetes in 15 (16.9%), family history in 15 (16.9%) and obesity in 18 patients (20.2%), respectively.

### CMR results

**Fig. 1:**
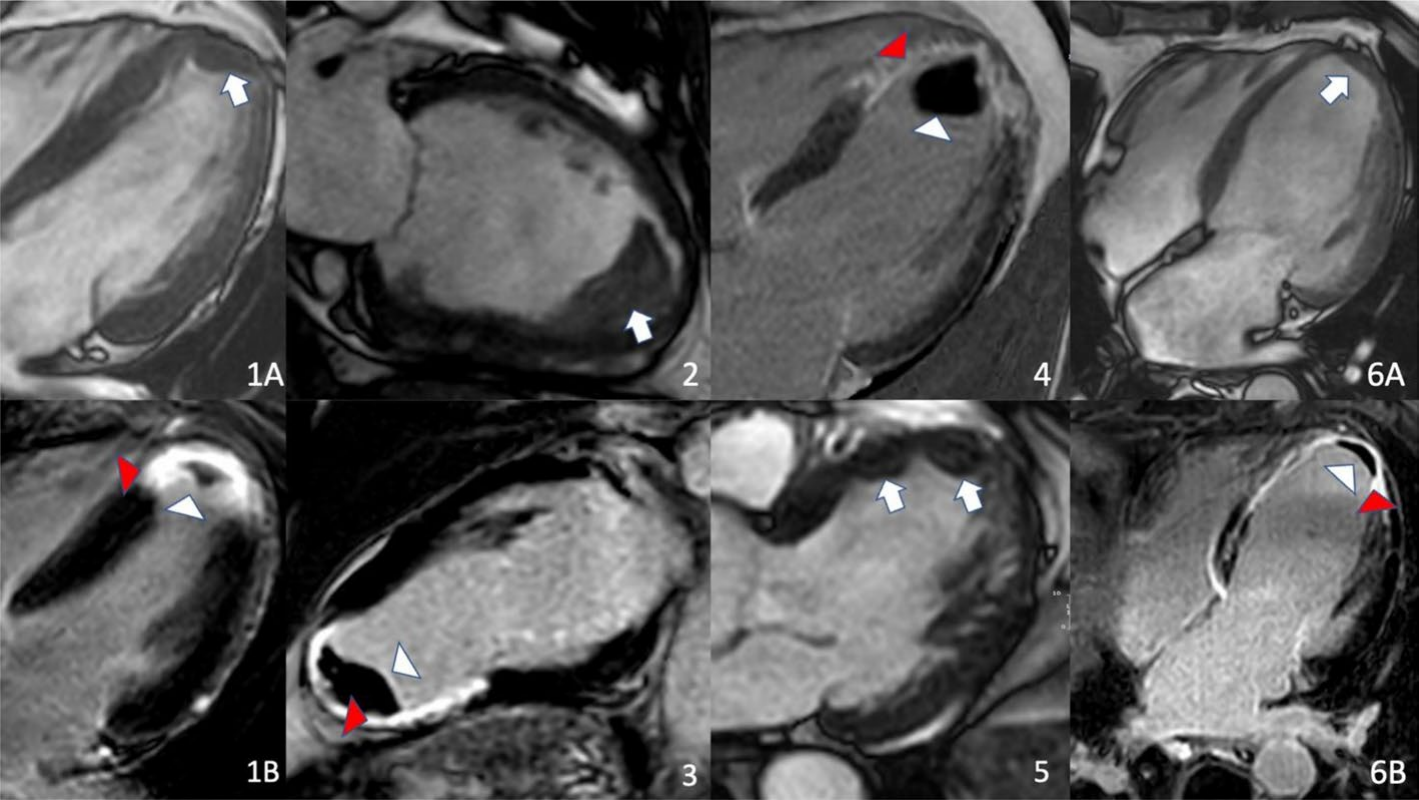
Image examples of six patients (1–6) with an LV thrombus on cine images (arrows) or late gadolinium enhancement images (white arrowheads). Detection of a thrombus may be difficult on cine images in some cases but is much easier on Late Gadolinium Enhancement images. Extensive transmural myocardial scar can be seen (red arrowheads) as predisposing factor for thrombus development. Patients either received high-dose dobutamine (1,3,6), low-dose dobutamine (2), or vasodilator stress with adenosine (4,5)

CMR showed a reduced ejection fraction in most patients (89.5%), with 46.3% of patients exhibiting an ejection fraction of < 40%, a known precipitator of thrombus development [43]. Global longitudinal strain was − 9.3 ± 2.2%, whereas regional longitudinal strain at the thrombus location was significantly lower (− 2.8 ± 2.6%, *p* < 0.001). In 87.4% of patients, the thrombus was located in the apex and local akinesia was present at the site of the thrombus in nearly all patients (98.9%). 83.2% had an aneurysm at the site of the thrombus. Multiple thrombi were present in 12.6% and protruding thrombi in 51.6%. The mean thrombus size (2D) was 1.6 ± 1.6 cm^2^. 43 patients underwent dobutamine stress (36 high-dose, 7 low-dose) whereas 52 underwent vasodilator stress CMR. Heart rate increased in all groups, most notably during high-dose dobutamine stress (Δ 68/min). The dobutamine stress CMR was positive in 3 cases and vasodilator stress CMR in 9 cases. The positive stress CMR lead to a successful revascularization of the affected coronary via PCI in 7 patients and via Bypass in 2 patients. In 1 patient, a coronary artery bypass surgery was initiated but ultimately failed. In 4 patients (2 of those with negative stress CMR but persisting symptoms) a revascularization via PCI was planned but estimated as technically infeasible during invasive diagnostic angiography. CMR data is given in Tables 2, 3 and exemplary cases are shown in Fig. 1.

**Table 2:** Overview of the Cine-derived cardiac measurements of all patients

	Unit	Mean	SD
LVEDD	mm	56.4	7.9
LVESD	mm	40.4	10.0
LVEDV/BSA	ml/m^2^	107.9	30.1
LVESV/BSA	ml/m^2^	67.0	28.7
LV-EF	%	39.6	9.4
Global longitudinal strain	–%	9.3	2.2
Regional longitudinal strain on thrombus location	–%	2.8	2.6
MAPSE	mm	9.2	2.5
Septal wall thickness	mm	12.0	2.1
Lateral wall thickness	mm	7.2	1.6
LV mass/BSA	g/m^2^	69.0	15.3
RVEDD	mm	43.9	6.9
TAPSE	mm	18.5	5.0

*LV* left ventricle, *LVEDD*  LV end-diastolic diameter, *LVESD* *LV* end-systolic diameter, *LVEDV  LV* end-diastolic volume, *BSA* body surface area, *LVESV LV* end-systolic volume, *LV-EF LV* ejection fraction, *MAPSE* mitral annular plane systolic excursion, *RVEDD* right ventricular end-diastolic diameter, *TAPSE*  tricuspid annular plane systolic excursion

**Table 3:** Overview of the performed stress exam including the type of stress agent, dosis of stress agent and vital signs before and during peak stress

	High-dose dobutamine	Low-dose dobutamine	Vasodilator stress
Number of patients	36	7	52
Stress medication	Dobutamine/atropine	Dobutamine	Adenosine^a^
Vital parameters at rest			
Heart rate (min)	67 ± 9	64 ± 13	67 ± 11
Systolic blood pressure (mmHg)	123 ± 17	118 ± 25	123 ± 16
Diastolic blood pressure (mmHg)	71 ± 12	68 ± 12	71 ± 9
Vital parameters at preak stress			
Heart rate (/min)	135 ± 14	84 ± 19	84 ± 13
Systolic blood pressure (mmHg)	129 ± 27	119 ± 19	115 ± 16
Diastolic blood pressure (mmHg)	68 ± 17	64 ± 9	61 ± 12
Stress result positive	3	n.a	9

^a^1 patient received vasodilator stress using a 0,4 mg single injection of regadenoson

### Safety of stress CMR in LV thrombus patients

Only three patients (3.1%) experienced moderate or severe symptoms during high-dose dobutamine stress, which completely resolved without medication. There were no minor or major complications during and up to 48 h after the CMR stress exam, and no examination had to be aborted. No patient in the control group experienced symptoms or complications during CMR and up to 48 h after the exam. Details on periprocedural safety data are given in Table 4.

**Table 4:** Overview of the periprocedural safety of stress CMRs and thrombus characteristics

Periprocedural complications	
Occurrence of symptoms alone (shortness of breath or angina)	3 (3.2%)
Non-tolerable symptoms with or without need for medication	0 (0%)
New arrhythmias	0 (0%)
New embolic event	0 (0%)
Other complications	0 (0%)
LV thrombus characteristics	
Size (2D), cm^2^	1.6 ± 1.6
Mobile	12 (12.6%)
Multiple thrombi	12 (12.6%)
Protruding into LV cavum	49 (51.6%)
Local acinesia	94 (98.9%)
Local aneurysm	79 (83.2%)
Apical location	83 (87.4%)

### Sensitivity of echocardiography and thrombus resolution rates

In 61 patients, multimodality imaging with an additional transthoracic echocardiography within 30 days was available. The LV thrombus was only found in 17 cases (27.9%) by echocardiography. As a results of the newly diagnosed LV thrombus, anticoagulation was initiated in 65 patients. Of those, only 11 LV thrombi would have been diagnosed with echocardiography alone and the diagnosis and treatment decision were solely dependent on CMR imaging in the other 54.

**Fig. 2:**
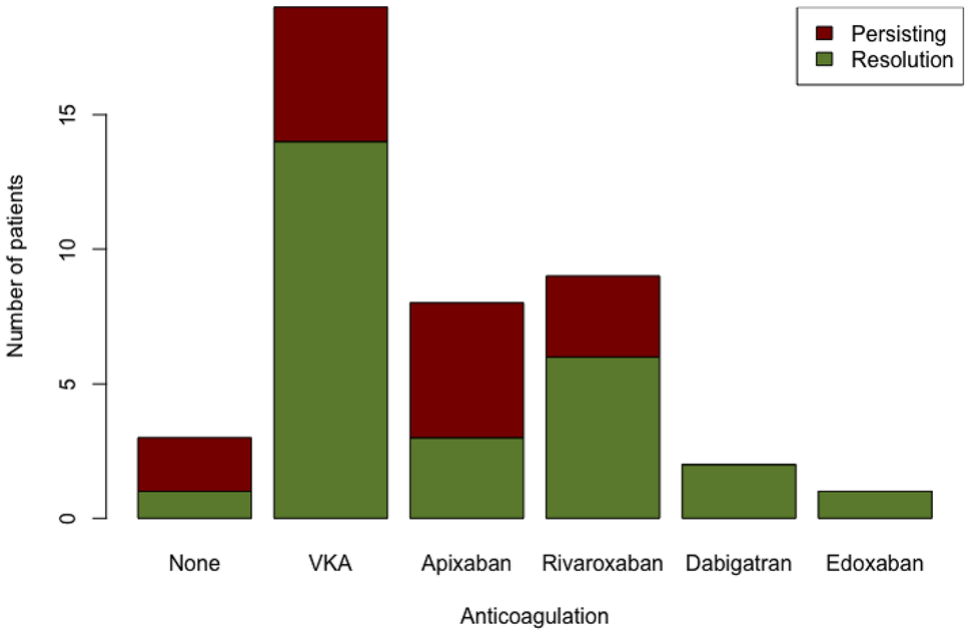
Thrombus resolution and choice of contrast agent in the study group. No statistical significance between choice of contrast agent and thrombus resolution was found

Follow-up imaging (echocardiography or CMR) was conducted for 74 patients at least 3 months after the initial CMR exam. A thrombus resolution was observed in 55 cases (74.3%). In the subgroup of patients undergoing CMR as follow-up, thrombus resolution was observed in 27 of 42 patients (64.3%). There was no significant correlation between the type of anticoagulant and thrombus resolution (*p* = 0.299). Data is displayed in Fig. 2.

### Outcome and embolic events before vs. after CMR

In total, 27 patients and controls experienced a MACE during the follow-up period (14.7%). There was no statistical difference between the occurrence of MACE between patients and controls (*p* = 0.45). Of those, 20 (9 in the study group, 11 in the control group) had occurred in the 12 months prior to the initial CMR exam with 12 strokes, 3 myocardial infarctions and 5 other arterial embolisms. During that time, only 29 (15.3%) of the study patients and controls were on permanent oral anticoagulation (12 on vitamin K antagonists (VKA) and 17 on direct oral anticoagulants). Among the 20 patients who experienced MACE before CMR, 15 were not receiving any anticoagulation.

**Fig. 3:**
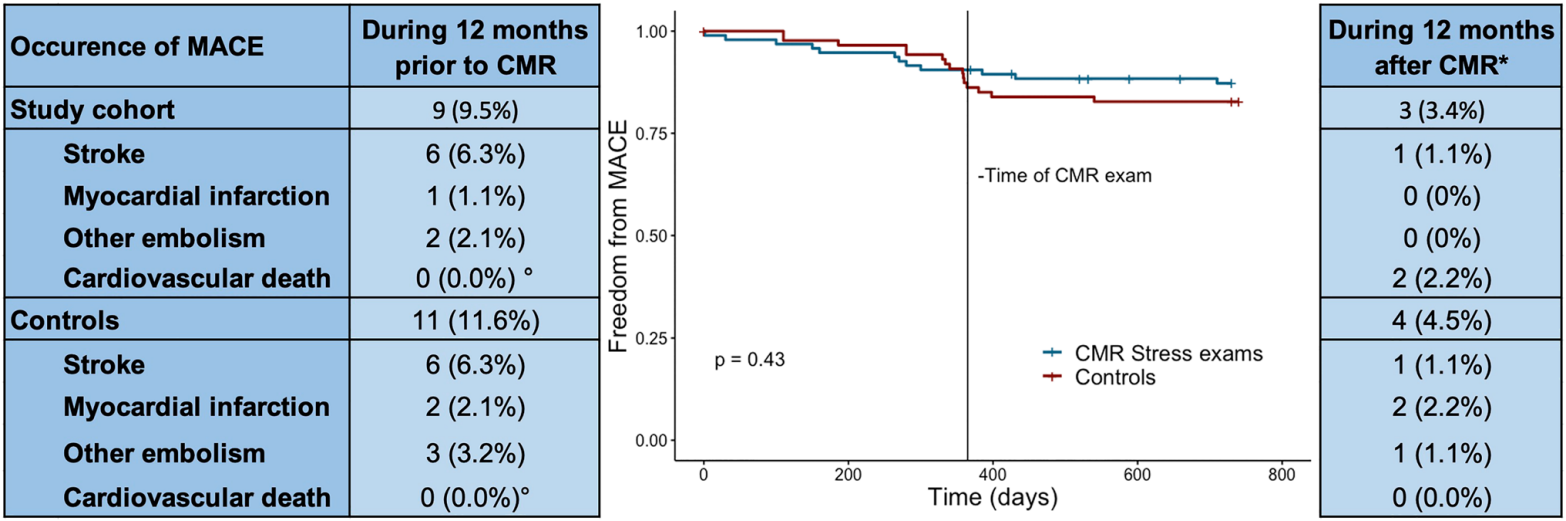
Event rates of MACE of the study group and an age-, sex- and ejection fraction-matched control group in the 12 months prior compared to 12 months after CMR. A Kaplan–Meier curve shows a graphical illustration of event rates for MACE with the vertical line indicating the time of CMR with subsequent diagnosis of LV thrombus and (in many cases) therapeutic changes. *Data available for 89 patients. °Per study design patients with death before CMR were not included in the study, therefore they are underrepresented and cannot be compared between groups

The thrombus characteristics size, mobility and protrusion did not correlate with the occurrence of embolic events in the study group (*p* = 0.625, *p *= 0.988, p = 0.848) or death (*p* = 0.329, *p* = 1, *p* = 0.236). Likewise, embolic events were independent of the chosen anticoagulant (*p* = 0.883 before CMR, p = 0.146 after CMR) and stress agent (*p* = 0.437) for the study group. During a median follow-up of 33.7 months, 6 deaths (6.3%) occurred in the study group with no correlation observed to the choice of stress agent or thrombus characteristics. The outcomes of the study group and controls are displayed in Fig. 3.

## Discussion

In a group of 95 patients with LV thrombi at a moderate to high cardiovascular risk, this study is the first to demonstrate a high periprocedural safety of dobutamine and adenosine stress CMR in the evaluation of CAD. No adverse event occurred during and up to 48 h after the exam. Nonetheless, patients were found to be at an overall high risk as 12 patients experienced a MACE during a follow-up of 24 months. However, this risk was not associated with the stress exam since no difference in outcome was observed when compared to a sex-, age- and left ventricular ejection fraction-matched control group without a stress exam. The addition of a stress test brought significant benefits to the patients, as 12 patients were scheduled for revascularization due to the stress exam results. In addition, there is less use of invasive diagnostic approaches after negative stress exams.

Our study reports an annual embolic event rate of 6.7% and an average annual mortality rate of 1.5%, which emphasizes the importance of thorough screening in high-risk patients [44]. Standard echocardiography alone demonstrated limited sensitivity detecting only 27.9% of LV thrombi in this study. Therefore, the identification of precipitating factors such as local wall akinesia or aneurysms through echocardiography should merit further investigations such as the use of ultrasound contrast agents or conduction of a CMR, if available [45]. In addition, cardiac computed tomography has been shown to possess high diagnostic accuracy and is widely available [46–50]. In patients with an indication for a contrast CT due to other comorbidities, cardiac CT may replace additional CMR imaging for further thrombus assessment.

A successful thrombus resolution after guideline-adherent therapy was observed in 64.3% to 74.3% of the study group, which underlines the importance of repeated imaging to adjust therapy [51]. The choice of anticoagulant did not affect resolution rates, adding evidence to previous data and current discussions about a comparable efficacy of direct oral anticoagulants and vitamin K antagonists [9, 21–24].

A higher rate of thrombus resolution might have been achieved through longer anticoagulation or by switching between anticoagulants in selected patients [2, 18, 21]. In this context it is of interest that 16.8% of the study patients were already receiving anticoagulation for other indications at the time of LV thrombus diagnosis, a fact that is not well established in the literature [52, 53].

Although the annual embolic event rate of 6.7% in our study is comparable to previous CMR studies, the reported annual embolic risk of previously published echocardiography studies on LV thrombus patients was higher at around 10% [25]. This difference may be attributed to the lower sensitivity of echocardiography in detecting thrombi, and therefore generally larger thrombus sizes in those studies [17, 18].

In contrast to previous data, protrusion and mobility of thrombi were not associated with an increased embolic risk. However, these studies were based on echocardiography at a time when PCI was not available as a treatment for myocardial infarction. Therefore, the comparability of these studies to our current findings may be limited [41, 54]. In agreement with our study, Cusick et al. reported no complications during moderate dobutamine stress echocardiography in 55 LV thrombus patients. However, the study fails to achieve an adequately high heart rate response (peak heart rate 114/min) and does not include postprocedural observations or outcome [55, 56].

Due to the design of our study, several limitations must be considered. Firstly, the retrospective and monocentric design of the study is a significant limitation. Together with the moderate-sized sample size it limits the generalizability and rare complications might be underrepresented. However, the study serves as an important initial investigation for the clinical use of stress CMR in appropriate LV thrombus patients and provides a basis for future prospective studies on this topic. Secondly, the embolic event rates of the study might be underestimated as small events might go unnoticed by the patients and treating physicians. Nonetheless, that is also applicable to most studies on the topic. Thirdly, the study only analysed CMR patients, which is not universally and timely available. Therefore, a selection bias needs to be considered when interpreting the data. Fourthly, the assessment of anticoagulation efficacy and an intramodality comparison to echocardiography were not the primary endpoints and thus, the statistical power is limited. In addition, the sensitivity of echocardiography might have been partly improved by the use of ultrasound contrast agents. However, the use of contrast agents is not part of a clinical routine in our hospital and was not done in our study patients. Lastly, our study only assessed drug-induced stress tests and new stressors like dynamic handgrip exercises or hyperventilation were not analysed [57–60].

## Conclusions

Despite a high risk of thromboembolic events in patients diagnosed with a LV thrombus, no periprocedural adverse events occurred during CMR stress testing with dobutamine or vasodilators. During a 12 month follow-up, the occurrence of MACE was not statistically different to a control group.

## Data Availability

The datasets used and analyzed during the current study are available from the corresponding author upon reasonable request.

## Abbreviations

CAD: Coronary artery disease
CMR: Cardiac magnetic resonance imaging
LV: Left ventricular
MACE: Major adverse cardiovascular event
SD: Standard deviation
PCI: Percutaneous coronary intervention
